# Young children's narrative retell in response to static and animated stories

**DOI:** 10.1111/1460-6984.12523

**Published:** 2020-01-11

**Authors:** Emily A. Diehm, Carla Wood, Jane Puhlman, Maya Callendar

**Affiliations:** ^1^ School of Communication Science and Disorders Florida State University Tallahassee FL USA

**Keywords:** assessment, language, technology

## Abstract

**Background:**

The format of narrative prompts used within language assessment has remained relatively constant; however, the use of animated video prompts deserves exploration given the increase in access to technology.

**Aims:**

To investigate the effect of story presentation format (static picture book versus animated video) on preschool children's narrative retells. It was hypothesized that children would produce more advanced narrative language elements in response to an animated video than a book.

**Methods & Procedures:**

A total of 73 children between the ages of 3 and 5 years completed two narrative retells. One of these retells was in response to a 3‐min animated video presented on a computer; the other retell was in response to static pictures presented in a book format. Children's stories were then transcribed and coded for linguistic and narrative elements.

**Outcomes & Results:**

Paired sample *t*‐tests revealed that children produced significantly longer stories, greater syntactic complexity and lexical variety, and more action verbs when retelling an animated story compared with a book. Furthermore, a post‐hoc analysis revealed that examiners provided significantly fewer prompts when eliciting the retell with animation.

**Conclusions & Implications:**

Typically developing children demonstrated higher quantity and quality of language within a story retell in response to an animated video than after viewing images from the video presented in a static picture book. Although the extent to which these findings may be similar for children with language disorder has yet to be determined, researchers or clinicians should consider the effect of elicitation procedures on children's retells.

**What this paper adds**

**What is already known on the subject**

Narrative retells are often included as a tool to help differentiate children with typically developing language versus those with a language disorder. Differences in narrative prompts (e.g., retell versus personal generation, visual support) are associated with quantitative and qualitative changes within children's narrative retells.

**What this paper adds to existing knowledge**

Given that technology is often readily available and of interest to children, we explored the possibility of eliciting young children's narrative retells with an animated video prompt as an alternative or supplemental elicitation context.

**What are the potential or actual clinical implications of this work?**

Typically developing children between the ages of 3 and 5 years produced longer stories with more complex language in their narrative retells after viewing a video compared with looking at static pictures. Speech and language therapists should consider the impact of multimedia features within narrative prompts on children's performance during an assessment.

## Introduction

Language sampling is essential to the study of child language development and disorders. Gathering a connected language sample is well established as best practice to support differential diagnosis and for ongoing language assessment and progress monitoring (e.g., Botting 2002, Fey *et al*. 2004). Language sampling has been used to study multiple aspects of language, particularly grammar, vocabulary and discourse (Miller and Chapman 2012), and has been among the most commonly used informal language assessment procedure for decades.

### Narrative elicitation and measurement

Narrative samples are important to include within a comprehensive evaluation due to the documented relationships between early oral narrative productions and later reading comprehension (e.g., Griffin *et al*. 2004, Roth *et al*. 1996), academic achievement (e.g., Fazio *et al*. 1996), and performance on norm‐referenced assessments of language (e.g., Ebert and Scott 2014, Scott and Windsor 2000). Narrative language samples are generally elicited using one of two tasks: (1) a story generation task, in which the child invents a fictional story or recalls an event that happened to him/her (e.g., Ebert and Scott 2014, Schachter and Craig 2013); or (2) a story retell task in which the examiner tells a story and the child retells it (e.g., Cowley and Glasgow 1994, Gillam and Pearson 2004). Traditionally, researchers have analysed children's narratives across two dimensions: microstructure and macrostructure. In a microstructural analysis, one conducts fine‐grained, linguistic analyses of vocabulary and grammar, often calculating linguistic measures commonly reported in language sampling analyses (e.g., number of total words, type–token ratio, use of simple, compound or complex sentences). The latter approach, macrostructural analysis, consists of a generalized, pragmatic analysis of the overall content of the story, including aspects of story grammar (SG; e.g., setting, character(s), initiating event, action, consequence, ending).

#### Narrative retells

In order to control the story content so that comparisons may be made across children, many commercially available criterion‐ and norm‐referenced assessments and tools use narrative retell prompts rather than prompts that allow personal generation (e.g., Renfrew Bus Story; Test of Narrative Language; Systematic Analysis of Language Transcript software normative databases). Beyond control of story content, retell prompts may also have an important effect of children's stories, with research suggesting that children produce longer narrative samples and include more macrostructure elements when retelling a story than when generating a personal story (Merrit and Liles 1987).

The manner in which the examiner presents the narrative prompt is also related to meaningful performance differences. For example, Schneider and Dubé (2005) examined the effect of presentation mode by presenting stories to kindergarten and second grade students in three conditions: (1) an oral model with no pictures; (2) a combined condition with an oral model and pictures; and (3) picture presentation only without an oral model. Their findings revealed a difference in performance based on age, as kindergarteners retold more story content after hearing the story in combination with the pictures. In comparison, the second grade students retold similarly detailed stories in response to both an oral model of the story and the combined oral–pictures story presentation, indicating a possible benefit of multimodal story components (i.e., hearing and seeing the story) for young children only. Both age groups recalled fewer story details when the story was presented via pictures only.

### Impact of multimedia features

In recent years, linguistic intervention and assessment studies have increasingly incorporated modern multimedia features (e.g., audio, animation, music, interactive content) and tools (e.g., iPad apps) to align with the interest of children in the technology age. One specific multimedia feature, animation, may present advantages for young children's language production, particularly on naming tasks (Bétrancourt and Tversky 2000, Schlosser *et al*. 2014). One such study found that children aged 3–5 years (*n* = 220) named animated symbols more readily than static symbols (Schlosser *et al*. 2014). Additionally, there was an interaction between format of the symbols (animated and static) and word class, in that the investigators found a greater advantage of animation on labelling verbs than prepositions. This finding suggests that children may demonstrate better recognition of actions when accompanied by animation; a finding similar to that previously reported by Mineo *et al*. (2008) who found that children increased identification of action verbs when the target items contained animation. Further, Verhallen *et al*. (2006) reported that children from families with limited educational attainment increased their linguistic and comprehension abilities to a greater extent when exposed to multimedia stories (i.e., stories that include video, sounds and music) than static books (also presented on a computer). The impact of multimedia features on word learning may be especially pronounced for children learning a second language (Verhallen and Bus 2010). Yet, despite the documented benefits described above for some populations, multimedia features may not provide advantages to all. In children with diagnosed language disorders, Smeets *et al*. (2014) found that presentation of story (i.e., static or video) did not impact children's ability to learn new words, with both conditions promoting similar amounts of word learning.

With respect to the use of multimedia features within narrative assessment, a couple of studies have incorporated multimedia features (e.g., recorded audio, sound effects) when assessing children's ability to generate or retell a story, act out a story with toys, or answer comprehension questions (e.g., Gazella and Stockman 2003, Gibbons *et al*. 1986). These studies included typically developing children in preschool (i.e., 4 and 5 years old; Gazella and Stockman 2003) or children across age ranges (i.e., 4‐ and 7‐year‐olds; Gibbons *et al*. 1986) and incorporated different multimedia features (e.g., researcher‐developed videos with manually manipulated puppets or ‘stop animation’). Although both of these studies investigated children's narrative language after listening to a story (audio only) or seeing pictures while listening to a story (audiovisual), Gazella and Stockman (2003) analysed microstructural aspects of children's narratives (e.g., number of total words, number of different words), while Gibbons *et al*. (1986) were interested in differential performance at the microstructural level (e.g., acting out a story, retelling a story). For preschool‐aged children, there were no difference in microstructural performance between audio and audiovisual conditions (Gazella and Stockman 2003). However, a difference was observed for macrostructure in 4‐year‐old children, as they made more inferences and told better stories when presented with the stories in an audiovisual format than in an audio‐only format. This difference was not found for older peers (i.e., 7‐year‐olds) though, suggesting that younger children may experience a particular benefit from audiovisual prompts before retelling or generating a story.

Despite the innovative ideas involving multimedia features tested in the intervention and assessment studies above, no study assessed the impact of multimedia features on children's narrative abilities at both the macro‐ and microstructure levels. Furthermore, many of these studies were conducted years ago, and as such included multimedia features that would now be considered outdated (e.g., use of ‘stop animation’ in Gibbons *et al*. 1986). Moreover, it is possible that when these studies were conducted, children may have had more exposure and experience with books than with televised or animated videos. Therefore, we have a limited understanding of how present‐day multimedia features may impact children's narrative retell performance, specifically the effect of animation, given its possible role in word naming and learning.

#### Book versus video presentation

To our knowledge, only one recent study investigated children's narrative performance in response to a physical book or video, and occurred outside the context of an intervention. Klop and Engelbrecht (2013) investigated the effect of presentation modality on narrative quantity and quality by presenting silent animated videos and wordless picture books to typically developing students who were in third grade, with 10 students in each condition (*n* = 20). The findings demonstrated no significant differences in the total number of words, lexical diversity or syntactic complexity between groups who received static (wordless picture books) and dynamic (animated video) presentations. Indeed, both static and animated elicitation presentations yielded comparable narratives for participants; although the authors pointed out the study was likely underpowered to detect significant differences with only 10 participants in each condition.

Although animated stories may be a promising practice for assessment and intervention, there are potential drawbacks. Given the paucity of research on stories presented in an animated video, there is a gap in clinical utility in that normative data for traditional story retell may be different than when the story is presented as an animated video. While related research described above suggests a potential advantage of animations for language, the rapid presentation of events in a video may result in a larger number of ‘missed’ events, possibly decreasing the likelihood of a detailed retell.

Given that digital technology influences children's learning and is increasingly more integrated into daily activities (Warschauer and Matuchniak 2010), it seems important to better understand how animation may affect young children's performance on a measure of narrative retell. Therefore, in the current project, we examined young preschool‐aged children's narrative language when presented with two retell presentation formats and asked the following questions:

- Do children's narrative retell productions differ at the microstructural level (e.g., number of total words, number of different words, type token ratio, mean length of utterance, number of action verbs) in response to a story presented as an animated video or static picture book?
- Do children's narrative retell productions differ at the macrostructural level (e.g., inclusion of character names, setting, problem, attempts, consequence, ending) in response to a story presented as an animated video or static picture book?

Considering available literature, we hypothesized that typically developing children aged 3–5 years would produce greater quality and quantity of language when the story was presented as a video with animation.

## Method

### Video and book development

In order to compare elicitation conditions (i.e., video versus book with static pictures), we wanted to keep the story content across the two conditions as similar as possible. When considering how to do this, we had two options: (1) use a book to develop a corresponding video; or (2) use a video to develop a corresponding book. Our team determined that the second option was the most feasible for the current project, and therefore we first located videos before developing their corresponding picture books.

#### Video selection

We located previously made videos online (e.g., YouTube) or through film school websites that were similar across seemingly important characteristics (e.g., developmentally appropriate content, engaging animation, non‐violent, 3 min, or so, in length). Videos with mainstream characters (e.g., Clifford) or mainstream animations (e.g., Pixar shorts) common to children in the United States were excluded from the study to avoid potential familiarity with characters, plot and story events. After previewing over 20 videos found online, we selected four videos for inclusion in the study—each of which had a relatively simple plot that followed the organization of a ‘classic narrative’ and contained at least one episode (i.e., problem/initiating event, attempt to solve the problem, consequence, ending), were approximately 2.5 min in length, did not contain violence and included a maximum of three characters. Additionally, we believed that the selected videos depicted story events and characters’ motivations and feelings without requiring much inference or prior background knowledge on part of the participant in order to comprehend the story.

#### Story scripts

After selecting the videos, we constructed four narrative scripts (see appendix A), similar in terms of length and semantic and syntactic complexity, to correspond to each of the four videos. The scripts were separated into terminable units (t‐units, consisting of the main clause (subject and verb) and any dependent clauses) and analysed using Systematic Analysis of Language Transcript (SALT; Miller and Chapman 2012) software. As seen in table 1, the stories varied somewhat in terms of overall utterance counts (i.e., number of t‐units), number of total words (NTW), number of different words (NDW) and mean length of utterance (MLU). Although there was linguistic variability among story scripts, the use of randomization to assign stories to participants was thought to minimize systematic differences in retell ability that may occur due to receiving a story with more complex (or simple) language. Additionally, because the main purpose of this project was to determine if video and book conditions elicit different narrative retells from children, significant findings between conditions despite variability in linguistic complexity or length of the story allows for conclusions regarding presentation condition to be made with even greater confidence. That is, the difference in narratives retold by children after exposure to a video and book is found not only for stories with a given MLU or NTW, but rather across stories of varying length and semantic and syntactic complexities.

**Table 1:** Descriptive statistics of story scripts

Story	Total number of t‐units	MLU	NTW	NDW	Animation/audio length (min:s)
*Billy Bear & The Balloon*	30	9.3	279	130	2:35
*You Can't Catch Me*	29	10.14	294	151	2:15
*Pingu Goes Fishing*	32	11.41	365	148	2:19
*That's My Hat*	41	8.95	367	158	2:35

Note: MLU, mean length of utterance; NTW, number of total words; NDW, number of different words. All stories contained at least three examples of each microstructure element (e.g., compound sentence, adverb, pluralized noun) scored on the NAP.

Given that the participants’ narratives would be scored on the inclusion of type and frequency of micro‐ and macrostructural elements, the four story scripts included at least three instances of each microstructure element of the Narrative Assessment Protocol (NAP; Pence *et al*. 2007), such as complex sentences and prepositional phrases. Each script also contained at least one example of the SG elements on our scoring rubric (table 2) to ensure similar macrostructural complexity across stories. The narrative scripts were audio recorded by the fourth author to ensure similar assessment experiences (e.g., same rate, pitch and timing of storybook reading) across participants. The recorded narratives were mapped onto each corresponding animated video for simultaneous audiovisual presentation using the program Sony Vegas Pro.

**Table 2:** Macrostructure scoring rubric: Codes and descriptions

Element	Description	Points possible
[C] Character	Includes the main character's name or a description of the main character; introduces characters in the story by *any* proper name or generic label (e.g., he, she) in an attempt to identify character(s)	1 point per unique character
[S] Setting	Tells where the story or events take place.	1 point per unique aspect
[P] Plot	Description of the activity that was occurring before the character's recognition of the problem/goal. May also include any dialogue that occurs before the initiating event	1 point per unique aspect
[IE] Initiating Event/problem/goal	Initiating events cause a response in the main character that sets the story in motion	Maximum of 1 point
[R] Reactions emotions	Thoughts/dialogue in response to problem/events	1 point per unique aspect
[A] Attempt	Attempt of the main character(s) to fix the problem	1 point per unique aspect, unless the same attempt occurred multiple times
[CO] Consequences	Explain what happened as a result of the attempt; what the secondary character does to help/not help the main character	1 point per unique aspect, unless the same consequence occurred multiple times
[E] Ending	Description of the event that occurs after the problem is fixed; a resolution of the problem/initiating event	1 point per unique aspect

#### Book development

To create picture books that corresponded to the videos, 18 screen shots (i.e., still frames) from each video, judged by the investigators to be the most indicative of the story's characters and events, were laminated and put into a double‐sided, spiral bound book for a total of nine pages per book. The books were presented with the same audio recording used within the animated format; however, the book audio contained chimes to indicate when to turn the page, ensuring similar viewing times per page across all participants.

### Participants

Participants were recruited from preschools or daycares within a mid‐sized Southeastern city in the United States. To be included, a participant was between 3 and 5 years of age, used spoken language as a primary means of communication, had sufficient visual and auditory abilities to listen to a story while watching a video/looking at a book, and spoke English during his/her school day, according to parent or teacher report. Parental consents and data were collected for 73 children enrolled in part‐ and full‐time preschool or daycare centres (25% and 75% of the sample, respectively). As shown in table 3, the majority of participants were 3 or 4 years old. Six 5‐year‐old children, whose birthdates were soon after the 1 September deadline for entry into kindergarten, were also included within the sample. Most children in the sample spoke English as a first language at home (93.2%); however, two children spoke Russian, one child spoke Chinese and one child reportedly spoke Japanese as their first language. These four non‐native English speakers spoke fluent English, as judged by the examiners, while completing all assessments. Parents indicated that 74% of participating children were white, 23% were African‐American, 5% were Asian and 4% were multiracial. The majority of mothers reporting having earned a bachelor's degree (43%). An additional 24.7% of mothers held a master's degree, 12.3% held an associate's degree, 5.5% completed some college but did not obtain a degree and 1.5% of the mothers held a high‐school diploma. Assenting parents of most children reported that their child was typically developing without sensory impairments or disabilities; however, parents of one female child reported a mild‐to‐moderate hearing loss in her right ear (for which she did not wear a hearing aid), and the parents of another female child reported a diagnosis of a developmental delay. Overall, participant performance on norm‐referenced measures (table 3) suggests that the sample was largely comprised of children with above‐average non‐verbal intelligence and auditory processing abilities. Unfortunately, a norm‐referenced measure of general language abilities was not included in the assessment battery due to time constraints.

**Table 3:** Demographic information and means (SD)

	All (*N* = 73)	3‐year‐olds (*n* = 30)	4‐year‐olds (*n* = 37)	5‐year‐olds (*n* = 6)
	F (*n* = 36), M (*n* = 37)	F (*n* = 13), M (*n* = 17)	F (*n* = 21), M (*n* = 16)	F (*n* = 2), M (*n* = 4)
	Mean (SD)	Mean (SD)	Mean (SD)	Mean (SD)
Age (months)	49.51 (8.06)	41.13 (3.23)	54.22 (3.47)	62.50 (3.53)
PTONI^a^	108.69 (18.88)	98.64 (19.32)	115.05 (15.15)	117.80 (19.46)
GRTR!‐R^b^	107.94 (13.45)	106.03 (16.28)	108.91 (11.24)	111.83 (9.43)
TAPS^c^	100.83 (20.84)^d^	n.a.^d^	104.37 (13.02)	102.60 (10.16)

Notes: F, female; M, male; PTONI, Primary Test of Nonverbal Intelligence; TAPS, Test of Auditory Processing Skills; GRTR!‐R, Get Ready to Read! ‐ Revised.

^d^TAPS provides standard scores for children ages ≥ 4 years; ^a^ *n* = 70; ^b^ *n* = 71; and ^c^ *n* = 68.

### Measures

Along with the investigators, trained undergraduate and graduate student researchers individually assessed participants’ non‐verbal intelligence, auditory memory and narrative retell ability. With respect to narrative assessment, all 73 participants completed two narrative retells: one after exposure to a story presented in a static picture book and the other in response to a different story presented as an animated video. Eight participants did not complete the norm‐referenced assessment battery in its entirety due to changing schools or multiple absences from school. Of these students, seven participants completed at least two of the three norm‐referenced measures. Measures for which there was missing data are reflected in table 3. Non‐standardized raw scores were used for all other analyses.

#### Non‐verbal intelligence

Given the comprehensive linguistic nature of the narrative measures included in the current study, we administered the Primary Test of Nonverbal Intelligence (PTONI; Ehrler and McGhee 2008) to assess participants’ non‐linguistic cognitive abilities. This measure uses pictures to assess reasoning ability (e.g., which picture does not belong) in young children without requiring a verbal response. This assessment has a mean score of 100 and a standard deviation (SD) of 15. Additionally, the authors of the PTONI report a coefficient alpha of 0.93, test–retest reliability of 0.97 and interrater reliability of 0.99 in the manual, indicating that the assessment has adequate reliability.

#### Auditory memory

We administered the Test of Auditory Processing Skills—3rd Edition (TAPS‐3; Martin and Brownwell 2005) to measure participants’ auditory memory, as the ability to listen and repeat words may impact one's narrative retell performance. We administered four subtests: (1) the Number Memory Forward subtest (*α* = 0.73–0.71) requires children to orally repeat number sequence; (2) the Number Memory Reversed subtest (*α* = 0.73–0.71) requires a child to repeat a number sequence in the reversed order in which it was spoken to him/her; (3) the Word Memory subtest (*α* = 0.63–0.66) requires children to repeat strings of words; and (4) the Sentence Memory subtest (*α* = 0.74–0.71), which requires children to repeat sentences of increasing length and complexity. This assessment has a mean of 100 and SD of 15. Administration and scoring followed the assessment manual guidelines. Three‐year‐old children, not included in the norming sample of the TAPS‐3 (4–18 years old), demonstrated difficulty answering the Number Memory Forward/Reversed items in a pilot study and therefore only received the Word Memory and Sentence Memory subtests. Therefore, summed raw scores for the Word and Sentence Memory subtests were used for analyses.

### Measures of narrative retell ability

#### Analysis of microstructure

We conducted several analyses of participants’ narrative retells by calculating microstructure performance as measured by (1) traditional measures language sampling; (2) the NAP (a formal criterion‐referenced assessment of microstructure); and (3) the number of action verbs produced in the retells.

##### Traditional measures of language sampling

Although microstructural analysis procedures vary, researchers commonly report measures of linguistic productivity, diversity and complexity. Within this investigation, we used NTW as a measure of linguistic productivity (e.g., Fey *et al*. 2004), NDW as a measure of semantic diversity (e.g., Miller 1991; Scott and Windsor 2000), and mean length of utterance in morphemes (MLUm) as a measure of syntactic complexity (e.g., Fey *et al*. 2004). There was considerable range in NTW for both conditions (Book NTW ranged from 0 to 160; Video NTW ranged from 0 to 162). There were six participants in each condition who did not say anything (i.e., NTW = 0 for six participants during the animated retell; NTW = 0 for six participants during the book retell); however, since these participants *did* tell a story with at least one word, in at least one of the conditions, we did not remove their scores from our data. We acknowledge the problematic nature of limited samples for precise language sampling measures, as common practice recommends at least 50 words. Therefore, we also calculated the type token ratio (TTR) for each child, as this measure considers the different words a child produces relative to the total number of words in the sample (i.e., NDW divided by NTW), with a TTR of 0.50 indicative of an appropriate amount of vocabulary diversity (Templin 1957, as cited in Retherford 2007). All measures were calculated using SALT software (Miller and Chapman 2012).

##### Narrative assessment protocol

In addition to measures of microstructure derived from ‘traditional’ language sampling, we scored each participant's narrative retell using the short form of the NAP (Pence *et al*. 2007) to assess sentence structure, phrase structure, modifiers, nouns, and verbs. Participants receive between 0 and 3 points on 12 elements (e.g., prepositional phrase, pluralized noun), with 1 point awarded for each inclusion of an element, not to exceed 3 points. Children's performance on the short form of the NAP has been found to correlate moderately (*r* = 0.34) with concurrent performance on a norm‐referenced measure of child language (CELF Preschool‐2; Semel *et al*. 2004) and the developers of the tool report an acceptable interrater reliability, with raters in agreement 93% of the time (Justice *et al*. 2010).

##### Production of action verbs

To determine if the inherent actions in the animated video condition increased child production of action verbs, we operationally defined and coded all verb tenses (i.e., past, present, future) of an action verb (i.e., any verb that represented something that a person, animal, or force of nature could do). We did not follow a specific list of actions to code, but rather coded any verb that conveyed an observable action.

#### Analysis of macrostructure

Analysis of macrostructure tends to be qualitative in nature, involving subjective ratings of the story content, organization and overall quality of components (Fey *et al*. 2004). Although the measures used for macrostructural analysis vary greatly in the existing research, we selected eight SG elements commonly measured in preschoolers’ narrative retells (Fiestas and Peña 2004, Schachter and Craig 2013): characters, setting, plot, initiating event/problem/goal, reaction/emotion, attempt, consequences and ending (for a description of each element, see table 2). To increase the coding accuracy of these elements, the first author analysed the script, video and pictures of the four stories and created a list of acceptable responses for each macrostructure element. Narrative samples with macrostructure language not on the example list, but considered acceptable by coders, were discussed with the first author and added to the rubric when an agreement was reached. With respect to scoring, 1 point was awarded for each inclusion of a SG element, with more than 1 point being possible in some categories (e.g., naming more than one character or including several examples of character dialogue in response to the problem). If, during the child's retell, the examiner provided the child with the main character's name as a prompt, the child was not awarded credit. Two macrostructure sum scores (book, video) were calculated for each participant.

### Procedures

Undergraduate and graduate students from a communication science and disorders program participated in multiple training sessions, led by the authors, to learn how to administer and score the above mentioned norm‐referenced assessments, as well as how to transcribe and code language samples according to SALT software (Miller and Chapman 2012) coding conventions. Research assistants met reliability requirements (agreement of 90% or higher) before scoring or coding participant data. One measure of microstructure employed within this investigation, the NAP (Pence *et al*. 2007), had training materials available through the developer's website (www.preschoollab.com) and were used by our research assistants to learn to reliably score children's narratives using this tool. The training process was similar to that used by the developers (see Justice *et al*. 2010, for a more detailed description of training).

#### Participant testing

Recruitment (e.g., flyers, consent forms distributed to local preschools) and involvement of participants was approved by the university Institutional Review Board. Once a child's parent(s)/caregiver(s) provided consent, the authors or trained research assistants administered assessments in a quiet space within a child's preschool or daycare centre. Children provided verbal assent for testing, which was generally completed over two 30‐min sessions occurring within at least 2 weeks of each other. All assessments were counterbalanced across participants to avoid an order or practice effect.

With respect to the narrative assessments, an examiner, using an ‘I do, we do, you do’ approach, guided each child through two practice narrative retells to ensure that all children were familiar with the act of retelling a story before the book and animated video prompts. In the first practice narrative retell (‘I do’), we played an audio recording of Benchmark Story 8, from the Test of Narrative Retell—Preschool (TNR‐P), a subtest of the Narrative Language Measures: Preschool (NLM:P; Spencer and Petersen 2012), on a laptop computer while the examiner and child looked at the accompanying sequence of five pictures. After the story ended, the pictures were removed and the examiner provided an accurate retelling of the story as a model. Similarly, for the second practice narrative retell (‘We do’), we played an audio recording of the script from TNR‐P Benchmark Story 5 (NLM:P; Spencer and Petersen 2012), while the examiner and child again viewed the corresponding picture sequence. However, this time, after the story ended, the child retold the story. The examiner supported children's retell if they had difficulty (e.g., examiner provided character names and important facts of the story) and encouraged children to continue if hesitations occurred (e.g., ‘What happened next?’). If a child omitted a significant event from the story, the examiner reminded the child of the importance of telling the ‘whole story’ and offered the omitted information. This retell was not scored, but rather served to familiarize the child with the task of retelling a story.

After the guided narrative retell was complete, the examiner then introduced the next story presented as either the static picture book or animated video (‘You do’). The order of these tasks, as well as which story (for both book and animated conditions) was presented, was randomized across participants to decrease the possibility of a practice effect on the subsequent narrative retell. We used a laptop computer to present the audio file containing the story for the static picture books, as well to present the animated videos. We standardized directions for the presentation conditions so that all participants heard the same directions, directing each child to retell the story to a stuffed animal, named Buster, who we used as an unfamiliar listener. Buster was introduced to the children before each presentation and was kept hidden until after each story was presented. If a child needed additional assistance after the initial prompt (‘Now you tell Buster what happened in that story’), the examiner provided one of the pre‐approved, standardized prompts for story initiation or continuation (table 4) as pilot testing (using a different sample of children) revealed that if a child did not remember the main character's name, it often prohibited him/her from starting to retell the story. Examiners used any of the allowable prompts, one time each (if needed), when a child remained silent for at least 7 s. When it appeared that the child was finished telling the story, the examiner said ‘Anything else?’ as a final prompt. Although examiners were aware of the study's primary hypotheses, they were unaware that their verbal prompts would later be counted and analysed (as a post hoc analysis) to determine if examiners attempted to elicit more language from children while retelling the video or book. We recorded participants’ video and book retell productions using an Olympus digital voice recorder, VN‐6200PC.

**Table 4:** Examiner prompts for book and movie narrative retell tasks

Prompts for initiation	Prompts for continuation
<Character's name> …	Tell me more
Once upon a time, <character's name> …	Then what happened?
Tell me about <character's name>	Umhmm/uhhuh
What happened in the story?	Tell me what you saw
Tell me anything you remember about the story	What else do you remember?
	Anything else?

#### Language sampling coding and analyses

Research assistants transcribed two narrative retell samples obtained from each participant (i.e., retells in response to book and video conditions) and coded each file according to the SALT (Miller and Chapman 2012) coding conventions. We separated utterances into c‐units (i.e., independent clauses and any associated dependent clauses) and only analysed complete and intelligible utterances. We also excluded child utterances that contained an exact repetitions of an examiner's utterance or comments judged by the research assistant to be off‐topic or unrelated to the story content (e.g., ‘I know the last page,’ or ‘Today is pyjama day’). Transcribers were not blind to presentation condition.

#### Interrater reliability

To ensure accuracy of scoring, two research assistants scored all norm‐referenced assessments. If the score calculated by the second research assistant did not match the score calculated by the first scorer, the second research assistant, with the assistance of the first author, sought to determine the reason for the discrepancy. We then used the corrected (if needed) score from the second research assistant for statistical analyses.

To determine the reliability of narrative transcription and scoring, the first author randomly selected 25% of the audio files (i.e., 18 book retells and 18 movie retells) and independently coded the files according to SALT coding conventions, action verbs, the short form of the NAP, and the macrostructure rubric. The average interrater reliability was 98.5% for c‐unit segmentation (range = 95–100%), 98.6% for marking bound morphemes (range = 90–100%), 95% for action verbs, 100% for mental state verbs, 100% for linguistic verbs, 96.3% for NAP short‐form elements (range = 92–99%), and 92.5% for the total score on the macrostructure rubric (range = 90–95%). Disagreements were resolved through discussion.

## Results

To determine if the modality of presentation, static picture book or animated video produced quantitative and qualitative differences in preschoolers’ narrative retell, we conducted a series of paired *t*‐tests. A Bonferroni correction was applied (0.05/8 = 0.006) to adjust for type 1 error rate and was used to determine significant differences in the two conditions. We also calculated measures of effect size (i.e., Cohen's *d*) as an indicator of practical significance. Cohen's *d*‐values of 0.2, 0.5 and 0.8 may be interpreted as small, medium and large effects, respectively (Cohen 1988). As stated, we hypothesized that the videos would elicit greater quantity and quality of language in a narrative retell and several practical and statistically significant findings supported this hypothesis.

As shown in table 5, several measures of microstructure were significantly greater in the narratives children produced in response to the video compared with the narratives told by participants after viewing the book. Specifically, children produced significantly more total words and different words in response to videos than books. Additionally, children's video retells contained more complex syntax, as MLUm was significantly greater in the animation condition. Small effect sizes were observed on each of these microstructural variables (*d*’s = 0.36–0.41). Despite the statistically significant differences observed between the two conditions on measures of microstructure derived from traditional language transcription analyses, we did not find a significant difference in children's mean NAP scores between conditions after applying the Bonferroni correction; however, a small practical effect was observed (*d* = 0.26) in favour of the video condition. We also examined the frequencies of action verbs within video and book retell conditions. Results indicated significantly higher production of action verbs in response to the animated video than the book, which also corresponded with an effect size of *d* = 0.42.

**Table 5:** Video and book retell means (SD) and paired sample *t*‐test results

	Condition				
	Video, mean (SD)	Book, mean (SD)	*t* (72)	*p*	*d*
# Different Words	24.52 (16.12)	19.30 (14.12)	3.45	0.001^a^	0.40
# Total Words	46.77 (39.41)	34.01 (31.49)	3.52	0.001^a^	0.41
Type Token Ratio	0.56 (0.03)	0.63 (0.03)	–2.24	0.03	–0.26
MLU (morphemes)	6.02 (2.56)	5.17 (2.82)	3.07	0.003^a^	0.36
NAP Microstructure Score	8.21 (6.05)	6.89 (5.37)	2.23	0.029	0.26
# Action Verbs	6.68 (5.30)	4.85 (4.40)	3.62	0.001^a^	0.42
Macrostructure Score	5.34 (4.00)	4.29 (3.16)	2.57	0.01	0.30
# Examiner Prompts	5.79 (3.21)	7.37 (3.91)	–3.21	0.002^a^	–0.38

Notes: MLU, mean length of utterance; NAP, narrative assessment protocol.

^a^ *p* < 0.006 (significant difference after applying Bonferroni correction).

With respect to macrostructure, we compared mean sum scores on the researcher‐developed measure of SG for animated video and book conditions. Although the average raw macrostructure score was larger in response to the video, the difference between the two conditions was not significant after applying the Bonferroni correction (*p* = 0.01). In terms of practical significance, a small effect was observed (*d* = 0.30) in favour of the video condition. A sum score was used in the paired‐sample *t*‐test; however, descriptive statistics for means, standard deviations and maximum point values for the eight SG categories are provided in table 6. To avoid further decreasing power for finding statistically significant results, we did not test for statistical differences among the SG subcomponents. Nonetheless, children's mean scores on each of the SG subcomponents were higher for narratives produced in response to the video.

**Table 6:** Descriptive statistics for macrostructure subcomponents

Subcomponent	Video, mean (SD)	Book, mean (SD)
Character	1.45 (0.91)	1.38 (0.84)
Setting	0.16 (0.41)	0.08 (0.28)
Plot	0.67 (0.88)	0.58 (0.88)
Initiating events	0.44 (0.76)	0.30 (0.64)
Reactions	0.29 (0.68)	0.16 (0.50)
Actions	1.07 (1.57)	0.85 (1.16)
Consequences	0.66 (0.96)	0.47 (0.71)
Ending	0.60 (0.74)	0.47 (0.93)

Note: Maximum score of 1 for initiating events. Point values are not constrained for other subcomponents.

Lastly, we explored differences between conditions that may explain why children produced longer and more descriptive narratives in response to videos. Although we did not initially plan to examine the number of examiner prompts across presentation conditions, we hypothesized that the frequency of prompts provided by examiners during the retell tasks may explain our previous findings that children produced longer and more detailed retells in response to the video. The first author coded children's transcripts for each examiner prompt. If an examiner gave multiple, consecutive prompts (i.e., two or more prompts provided within 7 s without a child response), only the first prompt was coded. If a child responded to an examiner's prompt and was then prompted again for additional information, each prompt was coded. To determine if examiners provided more prompts during the video condition (possibly explaining children's longer and more detailed retells), a paired‐sample *t*‐test was used to compare the number of examiner prompts across conditions. We observed a significant difference, with examiners providing significantly fewer prompts to children during the video retell, which also corresponded to a negative, small effect (*d* = –0.36) (table 5).

## Discussion

In response to the main purpose of this investigation, our findings suggest that typically developing young children may be expected to produce longer narrative retells (NTW), use more diverse vocabulary (NDW) and use more complex syntax (MLUm) when retelling a short video compared with retelling the same story presented in a picture book format. Additionally, examiners provided children with fewer continuation prompts in the video condition despite being unaware that their prompts would be analysed, suggesting that children more readily retold the video than the book. We did not find a significant difference in children's narrative microstructure as measured by the NAP or TTR between conditions. Likewise, we did not find a significant difference between the macrostructure components included in participants’ retells in response to the video or book.

With respect to how our findings relate to the results of others, there are several notable similarities and differences. Longer retells and more diverse vocabulary within a video retell is consistent with the work of Schlosser *et al*. (2014) who reported an advantage of animations over static pictures on children's naming and verb labelling. The current results are also consistent with previous studies that observed a benefit of animations, including the findings that children learned and retained more vocabulary after exposure to an e‐book with animation and cinematic techniques (i.e., zoom, pan and edits; Verhallen and Bus 2010) or e‐books that included animation, background music and sounds of important story events (e.g., crackling sound of a fire; Smeets and Bus 2014). Taken together, the current findings substantiate a potential advantage of animation for children's retell in terms of quantity of words and number of different words in the retell.

However, the results from the current study differ from the findings of other research studies, specifically that conducted by Klop and Engelbrecht (2013), to which we offer several potential explanations. First, Klop and Engelbrecht's study may not have had sufficient power to detect significant differences between the conditions, as there were only 10 children in each condition. Second, their sample consisted of children between the ages of 8 and 9 years, whereas the present study's participants were about 5 years younger. Older students may have already developed the skills necessary to tell an acceptable narrative without actualizing support from visual input (be it animation or static pictures), thereby not demonstrating performance differences between the two conditions. It is possible that younger children, such as those in the current study, still benefit from the additional support provided by animation in the video; a benefit that exists beyond the visual support provided from static pictures. Lastly, although the differences mentioned above offer explanations based on participant or statistical characteristics, differences in methodology and story format may also have resulted in different results. Klop and Engelbrecht developed an original wordless picture book and associated video using a professional illustrator and graphic designer, respectively. Unfortunately, our research team did not have access to these professional supports, so we used previously created videos, as that was an ‘easier’ starting point than developing a video from a previously created book. Given that the story content was originally intended as a video, it is possible that we unintentionally introduced a bias towards the video condition. As such, we may have found different results had we designed materials in the same manner as Klop and Englebrecht, thereby removing any potential bias favouring the one condition over the other.

We also observed that children produced more action verbs in response to the video than in response to the book condition. Although the true cause or nature of the differences in action verb frequency cannot be deduced from the current study, it seems reasonable to expect that the video provided participants with increased transparency of action or access to the dynamic movements of characters and/or objects. If this is true, and actions were more salient in the video condition than in the book condition, it would logically follow that children may be more likely to remember and include the actions in their narrative retells following a video prompt. In order to truly test this hypothesis, however, we recommend that future studies use a book containing professionally drawn illustrations (as was done in Klop and Englebrecht) rather than those captured from ‘screen shots’ while watching a video—and code children's narratives for verb types.

The post‐hoc finding of fewer examiner prompts during the animated condition indicates that participants required fewer prompts when retelling the story when presented as a video than when presented as a book. Although we did not set out to measure the number of examiner prompts (and thereby provide different frequencies of prompts between conditions), it is plausible that children displayed increased attention or better comprehension of the story when viewing it as a video. Again, causal relationships or the nature of the difference in the number of examiner prompts cannot be deduced from the current design. However, because we counterbalanced condition presentation, and assigned stories to participants at random, any differences in examiner prompting are not thought to be related to child familiarity with the retelling task, but possibly instead to child engagement and eagerness to tell the story. Based on anecdotal notes and observations made by members of the research team, children appeared more enthusiastic and motivated to retell the video; however, we did not collect measures of participants’ attentional abilities, enthusiasm, or preference that would be required to support or defend these anecdotal reports.

### Limitations

The results should be interpreted cautiously in light of several limitations of the current study. Although we examined the difference in children's narrative retell performance using static and animated stories, we cannot draw conclusions regarding the specific features that accounted for these performance differences. It is beyond the scope of the study to distinguish the role of potential moderators such as engagement, movement or speed of action. Another potential limitation is that the static pictures were comprised of splices or screen shots of the animation. The importance and selection of 18 frames per story may not necessarily have reflected the frames that children would have selected as the most engaging or meaningful. Furthermore, had we first selected books and then developed corresponding videos, it is possible that children may have produced higher quality and quantity retells in response to the book condition, giving that we would be using a book in its original form.

### Implications

Despite limitations, these findings have implications for language assessment with young children. When gathering a narrative sample from a child between the ages of 3 and 5 years, the findings of this study suggest that clinicians should be mindful of how prompt format may affect a child's results. It is possible that within clinical populations, the use of animated narrative prompts may result in a greater quantity of language for analyses—similar to the results found in the current study. However, given that our current sample consisted largely of children with above average abilities from well‐educated families, the extent to which a similar finding may be observed in children with language disorder or from lower socio‐economic status backgrounds is unknown.

### Future research

This study should be replicated with children with identified language learning disorders and children with other disabilities. Because language samples are often used as a part of eligibility decision‐making or for progress monitoring of children receiving speech–language services, further investigation of the effects of animation is warranted with specific populations of interest to speech–language pathologists. The potential effect of animation may be different for children who differ in language abilities.

It would also be informative for follow‐up studies to isolate specific features to identify those that may contribute to the effect of animation or presentation condition (e.g., static pictures presented within a physical book versus an e‐book). The inclusion of comprehension questions after each presentation condition may also provide interesting insights on the nature of animation. After identifying mediators and moderators, additional studies are needed to examine the effect of narrative animation within intervention. Although intervention was not the focus of this study, it is possible that features found to be advantageous may also provide support or feedback during instruction.

## Appendix A Story scripts for four narratives

### You Can't Catch Me

On a warm summer night, a little firefly named Spark floated through the night's sky. ‘That looks like a friendly house,’ he thought, and flew towards the lights in the distance. Spark buzzed and flew through the broken screen to get inside the house. Spark didn't see the spiky‐tailed creature who also entered the house with him. Allie the Alligator saw Spark fly in the house so she followed him into the house, past the chair and sofa. Allie wanted to catch Spark and make him her pet or friend. Allie was sneaky and jumped and leaped around the room trying to catch Spark, but Spark was fluttering and moving too quickly. ‘Who is trying to catch me? I must fly faster!’ said Spark. Spark flew into the kitchen, barely escaping Allie's hands. ‘He's flying too high,’ thought Allie, ‘If I could only get closer to the ceiling!’ Allie climbed up on the chair and table and jumped off, reaching for the light. She became very disappointed when the electricity suddenly turned off. Allie thought Spark had escaped. But just then, she saw the firefly's bright light again. Allie lunged across the room and clasped the firefly in between her hands. ‘Oh no! What happened to your light, little firefly?’ said Allie. She threw the firefly, hoping he would begin flying again but Spark didn't fly. He only fell to the cold, hard, floor. Allie was upset until she saw Spark's light come back on. Allie realized that it was not nice to keep him as a pet, because Spark needed space to fly. ‘Do you want to fly little firefly? Fly away and play with your friends!’ ‘Thank you!’ replied Spark and he happily flew off into the dark night sky.

### Billy Bear and the Balloon

Once upon a time in a park, Billy Bear was walking and eating an ice cream cone, when he saw a hot air balloon in the sky. Wow! He was so excited about the balloon that he wasn't watching or paying attention to where he was going and he bumped into the trash can. Billy Bear searched and found his ice cream on a penguin's head! Oh no! That made Penguin Pete really mad! ‘Did you drop this ice cream on me?’ he yelled. Penguin Pete accidently let go of his balloon while he yelled at Billy Bear. His balloon went up into the sky toward the hot air balloon. Just then, Billy Bear got an idea! He saw Lizzy the Lizard selling balloons. Billy Bear bought a bunch of balloons to try to float in the sky like a hot air balloon. He tied the balloons to the trash can, to ride in it, but that didn't work. He tried to get more balloons but the wind blew strongly and they floated away. Then Billy Bear got stuck in the trash can and he couldn't get out. He was frustrated at first but then he got another idea. ‘My, what a big balloon!’ Then he got a much larger balloon and tied his trash can to the bottom of it. Billy Bear floated up a little bit but not quite far enough. He was still too heavy! When he realized that he couldn't soar, he began to throw things out of the trash can. ‘Hmm, what can I get rid of?’ he thought to himself. He threw out a heavy concrete block and then found the penguin in the trashcan! ‘What are you doing here silly penguin?’ said Billy Bear. He quickly tossed out the penguin and finally floated up into the sky. Off he went!

### That's My Hat

One sunny day, Felix was happily taking a nap in a hat he found lying on the floor. Just as he began to dream about catching a huge mouse, Joe, the owner of the hat, snatched the hat and put it on his head. Felix was not happy about having his nice bed being taken away. He wanted that hat back so he could finish his nap. Felix and Joe couldn't use the hat at the same time, so Felix had to get it back from Joe. Felix jumped up onto shelves above Joe's head, and tried to take the hat back. Joe was too quick! He grabbed the hat from Felix and put it back on his head. Felix jumped onto Joe's face but he was quickly pulled off and put on the ground. ‘I'll get him. What can I use? ‘ thought Felix. He looked around and saw tennis balls on the floor. Felix grabbed them and threw them at Joe. ‘Argh!’ yelled Joe. He grabbed Felix and threw him into the closet. ‘That will get him out of the way.’ But Joe did not know that Felix was a tricky cat. Felix snuck out of Joe's closet and hung on the ceiling above Joe's head. As Joe walked by, Felix used his sharpest claws to scratch Joe. ‘I have had enough!’ said Joe, so he grabbed the water bottle and sprayed cold water at Felix. ‘Will that make him stop?’ As Joe opened the door to leave, Felix was waiting for him in the entrance. Felix was ready with a hose. Felix soaked Joe and Joe shoved the door shut. ‘Phew!’ thought Joe. But oh no! Felix was on Joe's head! Felix grabbed the hat back and ran to the bedroom. ‘Ah finally’ thought Felix. ‘Now I can sleep,’ and he peacefully laid back down in the hat. Joe really wanted his hat back, so he got a cage and quietly snuck behind Felix. Felix was not a fool! He heard Joe coming. Felix fought back and put Joe in the cage! Do you think he was done? Nope, not yet! Felix grabbed the water bottle and squirted the helpless Joe. ‘Finally!’ thought Felix. ‘I can finish my cat‐nap.’

### Pingu Goes Fishing

One day Pingu went ice fishing on a frozen lake. He caught his first fish then he set out his line again with some lettuce to try to catch another one. Pingu waited and then he felt a few tugs on the line. This time while Pingu wasn't paying attention, Sully the seal who lived nearby came and grabbed the fishing line. Sully swam back to the other hole in the ice climbed out and placed the same fish that Pingu caught back on the line. ‘Hee hee hee,’ he laughed to himself, ‘Pingu has no idea!’ Again Pingu sat down to fish. Sully swam back over, pulled the line out of the other hole and began to eat the lettuce again. But this time, Pingu caught him. He saw Sully holding his fishing line and eating his lettuce. Pingu who was really angry yelled at Sully saying, ‘Hey! Why are you stealing my fish?’ Sully ran and jumped back into the water and quickly threw a snowball at Pingu before hiding back into the water again. Pingu tried but he couldn't catch Sully. He stopped a moment and thought to himself, ‘If I cover up one of the holes in the ice, could I make Sully come out of the other one?’ So Pingu covered up one of the holes and placed some yummy lettuce on the ice as bait for the Seal. Then he hid so that the seal would think that it was safe to come out. Just as the seal sneakily climbed out of the water, Pingu closed up the other hole so that he couldn't escape. Sully was trying to get back to the water and got his paw stuck under the heavy sheet of ice. It really hurt so he started to cry. ‘There, there,’ said Pingu, while holding his hands. Pingu felt bad so he let Sully go back into the water. Sully felt better. He gave Pingu back his fishing rod and said, ‘Would you like another fish?’ Then Sully gave Pingu a huge fish. It was much bigger than all of the other fish he caught that day so Pingu was very happy and he and Sully became very good friends.
